# A Word-of-Caution Against Euglycaemic DKA!

**DOI:** 10.5152/TJAR.2022.21144

**Published:** 2022-12-01

**Authors:** Rohan Magoon, Jes Jose

**Affiliations:** 1 Department of Cardiac Anaesthesia, Atal Bihari Vajpayee Institute of Medical Sciences, Dr Ram Manohar Lohia Hospital, New Delhi, India; 2 Department of Cardiac Anaesthesiology, Sri Jayadeva Institute of Cardiovascular Sciences and Research, Karnataka, India

Dear Editor,

Diabetic ketoacidosis (DKA) (blood glucose >250 mg dL^-1^, acidemia [venous pH <7.3 or bicarbonate <15 mEq L^-1^] and ketonaemia) is classified as an important perioperative complication. Moreover, an ever-evolving field of diabetology and the concomitant polypharmacy present additional challenges in the sound perioperative management of these metabolic disorders. This is epitomised by the resurgence of interest in euglycaemic DKA (EDKA). 

The data from the Food and Drug Administration (FDA) adverse-event reporting systems reveal that a sizeable percentage of the DKA patients (as high as 35% in certain scenarios) demonstrates blood glucose levels <200 mg dL^−1^.^1^ This DKA subset also referred to as EDKA manifests a triad of high anion-gap metabolic-acidosis, ketonaemia, and a near-normal blood glucose. The entity presents a significant diagnostic dilemma as euglycaemia may not result in a suspicion of DKA, thereby precluding an institutional DKA resuscitation protocol.^1^ This highlights the limitation of usual reliance on isolated sugar monitoring as a DKA screening tool, supporting the proposition of routine monitoring of ketonaemia in diagnosing perioperative diabetic crisis.^2^ Moreover, the related clinical features such as nausea, vomiting, tachycardia, hypotension, abdominal pain, and/or altered mentation can be confusing to interpret in the postoperative period predisposed to an enhanced catabolism. Nevertheless, subsequent to the diagnosis of EDKA, it is treated on the lines of DKA, with due care to avoid hypoglycaemia and a treatment target of a serum bicarbonate ≥ 15 mmol L^−1^, an anion-gap ≤ 12 mmol L^−1^, or a venous pH > 7.3.^3^

The usual triggers associated with EDKA are infection, starvation, vomiting or diarrhoea, and cocaine overdose. Recent reports of heightened EDKA risk with the perioperative use of sodium-glucose cotransporter-2 (SGLT2) inhibitors or gliflozins has captivated attention, particularly with these agents being increasingly employed as second-line hypoglycaemics.^4^ The commonly used gliflozins include canagliflozin, dapagliflozin, and empagliflozin. Their clinical effects are primarily due to inhibition of sodium-glucose transport protein 2 resulting in reduced renal glucose reabsorption and glycosuria leading to euglycaemia.^5^ This class of agents came into the limelight due to their purported additional benefits like weight loss, reduction in incidence of heart failure independent of glycaemic control, improved renal outcomes, and hepatoprotective properties in non-alcoholic steatohepatitis.^6^

However, a prominent case series by Lau et al^4^ outlined an accentuated risk of perioperative EDKA associated with gliflozins. The aforementioned in conjunction with other recent incriminating literature led to an updated FDA prescribing information in the march of 2020 with canagliflozin, dapagliflozin, and empagliflozin to be withheld for at least 3 days prior and ertugliflozin to be withheld at least 4 days prior to an elective surgery.^7^ The American Association of Clinical Endocrinologists and American College of Endocrinology also recommend that gliflozins be withheld preoperatively or in other situations with typical DKA triggers, like severe infection.^8^

Euglycaemic DKA is a rare clinical entity wherein a high degree of caution must be exercised. With ever growing number of diabetics and frequent use of gliflozins, EDKA incidence is bound to increase. Fasting patients with history of gliflozin use should be evaluated for high anion gap metabolic acidosis and ketonaemia despite having controlled blood glucose to unmask an underlying EDKA, thereby reducing mortality and morbidity. This highlights the fact that the pharmacodynamic, pharmacokinetic, and safety profile of the prescribed hypoglycaemics warrants due consideration in the conduct of safe anaesthesia.
